# Persistent and energetic bottom-trapped topographic Rossby waves observed in the southern South China Sea

**DOI:** 10.1038/srep24338

**Published:** 2016-04-14

**Authors:** Yeqiang Shu, Huijie Xue, Dongxiao Wang, Fei Chai, Qiang Xie, Shuqun Cai, Rongyu Chen, Ju Chen, Jian Li, Yunkai He

**Affiliations:** 1 State Key Laboratory of Tropical Oceanography, South China Sea Institute of Oceanology, Chinese Academy of Sciences, Guangzhou, People’s Republic of China; 2 School of Marine Sciences, University of Maine, Orono, Maine, USA; 3 Sanya Institute of Deep-Sea Science and Engineering, Chinese Academy of Sciences, Sanya, People’s Republic of China

## Abstract

Energetic fluctuations with periods of 9–14 days below a depth of 1400 m were observed in the southern South China Sea (SCS) from 5 years of direct measurements. We interpreted such fluctuations as topographic Rossby waves (TRWs) because they obey the dispersion relation. The TRWs persisted from May 24, 2009 to August 23, 2013, and their bottom current speed with a maximum of ~10 cm/s was one order of magnitude greater than the mean current and comparable to the tidal currents near the bottom. The bottom-trapped TRWs had an approximate trapping depth of 325 m and reference wavelength of ~82 km, which were likely excited by eddies above. Upper layer current speed that peaked approximately every 2 months could offer the energy sources for the persistent TRWs in the southern SCS. Energetic bottom-trapped TRWs may have a comparable role in deep circulation to tides in areas with complex topography.

Bottom-trapped topographic Rossby waves (TRWs) have frequencies ranging from several days to hundreds of days, which require a bottom slope to exist^1^. Water columns are stretched and compressed as they cross isobaths over the sloping topography. Governed by the conservation of potential vorticity, water parcels attain cyclonic or anticyclonic relative vorticity as they move toward deeper or shallower portions, respectively, of a slope in the Northern Hemisphere. TRWs propagate with shallower water to the right in the Northern Hemisphere^2^,^3^ and can be significantly modified by stratification^1^,^4^.

Both observations and simulations have demonstrated that TRWs dominate the current variability below 1000 m in the northwestern Atlantic and the Gulf of Mexico^3^,^5^,^6^. Thompson and Luyten^7^ provided observed evidence that TRWs on the continental rise have periods of 1–2 weeks and horizontal wavelengths of 100–200 km. Based on long-term current measurements, Hamilton^5^ found that the TRWs propagate westwards with significant periods of 25, 45, and 100 days in the Gulf of Mexico. Hamilton and Lugo-Fernandez^8^ found that the deep current induced by TRWs is highly energetic with a maximum speed of 90 cm/s and characteristic period of 10–20 days.

TRW generation is usually associated with upper layer forcing, such as the Loop Current (a current that enters the Gulf of Mexico through the Yucatan Channel and leaves between Key West and Cuba) and eddies, via potential vorticity adjustment to the changing depth of the bottom or interface^5^,^6^,^9^. Numerical studies have shown that the Loop Current and anticyclonic eddies radiate energy to the deep ocean with 20–100-day periods; these are concentrated in a band across the northern Gulf of Mexico around the 3000 m isobaths^3^,^10^,^11^,^12^.

The South China Sea (SCS) is the largest tropical marginal sea and spans a region from the equator to 23°N and from 99°E to 121°E with an average depth over 2000 m. It connects to the Pacific Ocean through the Luzon Strait, which is a deep channel with a sill depth of about 2400 m. There are four groups of islands within the SCS: the Dongsha, Xisha, Zhongsha, and Nansha Islands, which constitute the complex topography in the SCS (Fig. 1a). In this study, we focused on the Nansha Islands, which consist of more than 230 islands and reefs, have rich fishing grounds, and contain significant oil and natural gas reserves.

Thought to be driven by the Luzon Strait overflow, the deep SCS water flows southwards and arises in the southern basin to form the SCS deep meridional overturning circulation and cyclonic horizontal deep circulation^13^,^14^,^15^,^16^. The residence time of water in the deep SCS is estimated to be about 30–100 years^16^,^17^,^18^. The relatively quick replenishment of the deep SCS water indicates strong diapycnal mixing^19^, which is usually thought to be the result of breaking internal waves associated with the complex bottom topography^20^,^21^,^22^. However, the presence of TRWs in the complex SCS topography has not been investigated, and the roles of TRWs with regard to diapycnal mixing and deep circulation are still unknown.

Based on reanalysis data, Shu *et al.*^16^ found strong upwelling of the deep SCS water along the 3000 m isobaths and in areas of complex topography. They further found wave-like propagations along the slope around the SCS basin and speculated that TRWs may contribute to the deep circulation and abyssal mixing in the SCS. However, they did not give details on the TRWs and did not quantify the intensity of the TRWs relative to the tides. In this paper, we present the current measurements taken below 1400 m in the Nansha Islands region of the southern SCS and the observed evidence and importance of TRWs in deep currents.

## Results

### Sub-inertial fluctuations in deep layers

Figure 2 presents zonal velocity profiles in deep layers at M1 from May 24, 2009 to August 23, 2013. The most obvious feature of the zonal velocity was the persistent, energetic fluctuations with the period around 10 days. The velocity oscillations were coherent with no apparent propagation in the vertical direction, but the magnitude gradually increased towards the bottom. The zonal current was generally eastward with a time-averaged value of about 1 cm/s, which was consistent with the cyclonically deep circulation in the SCS basin^14^,^15^,^23^. However, the amplitude of the velocity oscillations could reach more than 10 cm/s, which was one order of magnitude greater than the long-term averaged current. Although the intensity varied with time, fluctuations were observed throughout the 5 years of observations. The observed meridional velocity had the same fluctuation characteristics except that it had a slightly smaller amplitude than the zonal current (Supplementary Fig. 1).

Figure 2e,f show the temperature at 1730 m (20 m above the bottom) and zonal velocity from the Aanderaa current meter at M1 from December 23, 2011 to August 26, 2012 and from August 27, 2012 to August 23, 2013, respectively. There were two types of strong fluctuations for both the bottom current and the temperature: the tidal signal and the sub-inertial frequency, which was slightly longer than 10 days. The bottom sub-inertial current had patterns similar to that observed by downward-looking acoustic Doppler current profilers (ADCPs). The time series of the 9–14-day band-pass filtered temperature and zonal velocity in Fig. 2e,f show that the temperature fluctuation lagged the velocity by about 3 days, which indicates a phase difference of about 90°.

In general, the data show persistent fluctuations in the deep current north of the Yongshu Reef in the southern SCS with a period of about 10 days and a depth-independent phase but bottom-intensified magnitude. These are typical characteristics of TRWs^24^,^25^,^26^.

### Features of TRWs

Considering the overlapping observation intervals of the Aanderaa current meter and downward-looking ADCP from December 23, 2011 to August 26, 2012 and from August 27, 2012 to August 23, 2013, these two periods form the main focus of our analysis below. Power spectra of the bottom velocities from the Aanderaa current meter at M1 are shown in Fig. 3. There were significant spectral peaks in the sub-inertial frequency band with a period of 9–14 days, which corresponded to the TRW frequencies described above. The frequency peaks were remarkable with peak values at 11.8 and 9.2 days during the first interval and 12 and 10.5 days during the second interval. The sub-inertial peaks in Fig. 3 indicated that the TRWs had a power density comparable to that of diurnal tides. It should be noted that there was a 15-day peak in Fig. 3a. When we used harmonic analysis to remove all tidal components, the 15-day peak disappeared, but the other sub-inertial peaks did not change. This indicated that the 15-day peak represented the spring neap tide and further confirmed that the TRW periods were 9–14 days. Thus, a 9–14 day band-pass filter was used to extract the TRWs from the ADCP and Aanderaa current meter observations.

The TRWs in Fig. 4c–f also suggest bottom trapping. For comparison, Fig. 4c–f show both the velocities associated with the TRWs and the tidal currents derived from the harmonic analysis. The amplitude of the tidal currents was ~5 cm/s. The TRW velocities had an amplitude comparable to that of the tide, and the former was slightly weaker most of the time. However, the TRW velocities were sometimes apparently larger than the tidal currents near the bottom. The largest amplitude of the TRW velocities at the bottom was 8.7 cm/s in the zonal direction and 6.5 cm/s in the meridional direction during March 2013. Thus, the largest amplitude of the TRWs was 11 cm/s near the bottom, which was about one order of magnitude larger than the time-averaged current.

**Figure 1:**
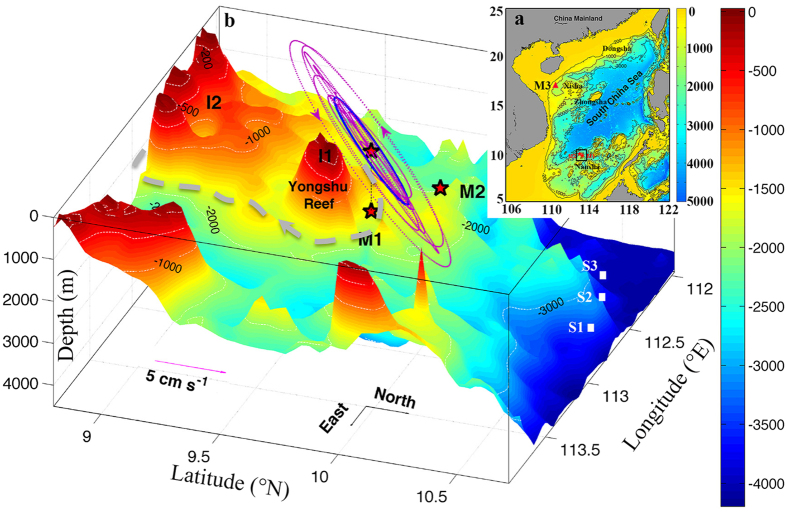
Bathymetry of the SCS. (**a**) The SCS topography; (**b**) three-dimensional zoomed in view of topography of the area inside the box in (**a**). The two stars in each subplot represent moorings 1 (M1) and 2 (M2). The triangle in (**a**) is an Aanderaa (M3) current meter moored in the Xisha islands area. The scatterplot (magenta dots) of zonal and meridional TRW velocities at 1730 m from 1 February to 30 May 2013 is shown in (**b**); it is projected to the surface plane for visual clarity. The blue line is the standard deviation ellipse of the TRW velocities. The “I1” and “I2” in (**b**) represent two islands. The three white squares denote the CTD stations from WOD, and the grey dashed line indicates a possible path of the TRWs. The colour maps indicate the water depth (unit: m). Maps were generated by using MATLAB.

**Figure 2:**
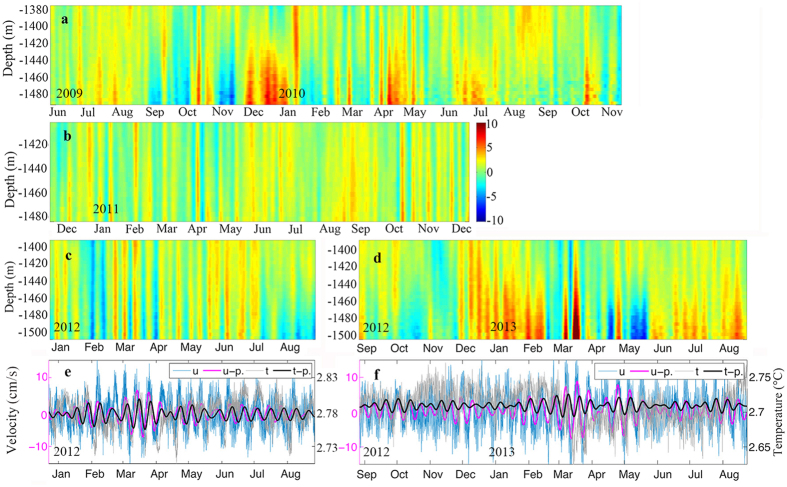
Deep-layer current observations. Observed near-bottom zonal velocity at M1 (unit: cm/s). (**a**–**d**) Time series of 3 day low-pass filtered zonal current profiles from the four segments of ADCP observations. Time series of the raw zonal velocity (cyan line) and temperature (grey line) obtained by the Aanderaa current meter at 1730 m (20 m above the bottom) (**e**) from December 23, 2011 to August 26, 2012 and (**f**) from August 27, 2012 to August 23, 2013. The solid heavy black and magenta lines represent the 9–14 day band-pass filtered zonal velocity and temperature, respectively. Figures were plotted using MATLAB.

**Figure 3:**
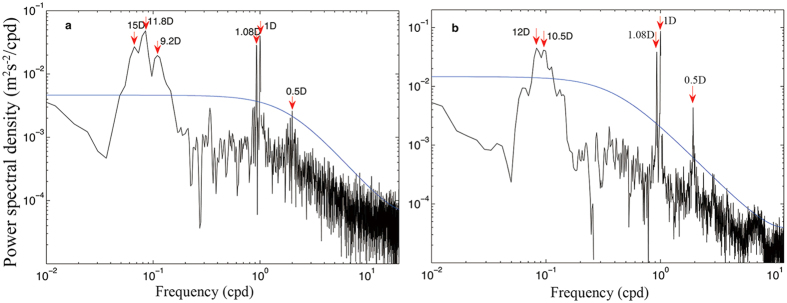
Power spectra of the bottom velocity. Power spectra of the zonal velocity near the bottom at 1730 m depth derived from M1 observations (**a**) between December 23, 2011 and August 26, 2012 and (**b**) between August 27, 2012 and August 23, 2013. Blue lines represent a significance level of 95%. Figures were plotted using MATLAB.

The scatterplot of the bottom TRW velocities from February 1 to May 30, 2013 shown in Fig. 1b (magenta dotted curve with the blue line representing the standard deviation ellipse) suggests that the water parcels rotated cyclonically. The major axis of the standard deviation ellipse of the TRW velocities was about 5 cm/s, and the angle between the axis and the zonal direction was about 37°. Under the assumption of *fα*/*H* ≫ *β*, where *f* is the local Coriolis parameter, *α* is the topographic slope, *H* is the water depth, and *β* is the gradient of *f*, the linear TRW theory^1^ predicts the following dispersion relation:


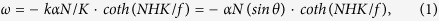


where *ω* is the TRW frequency, ***K*** is the wavevector with the components (*k*, *l*) in parallel and perpendicular to the bottom isobaths, *N* is buoyancy frequency, and *θ* represents the angle between the wavevector and the upslope direction or the angle between the velocity vector and isobaths^7^.

For short waves, *coth*(*NHK*/*f*) tends to 1, and eq. (1) becomes


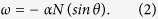


In this study, we derived *N* from three World Ocean Database (WOD) Conductivity Temperature Depth (CTD) profiles to be about 10^−3^ s^−1^ near the bottom, and the local slope was about 0.04. Because the topography is very complex, *θ* could not be accurately determined, but it fell in the range between 10 and 20 degrees. The estimated frequency from eq. (2) was approximately 1.10 × 10^−6^ to 2.18 × 10^−6^ Hz, which corresponded to a period of about 5.3–10.5 days. When we considered the topography at a slightly larger scale to account for the influence of both Island 1 (I1) and Island 2 (I2) (Fig. 1b), *θ* was a relatively small value of about 10°. Then, the inferred frequency was about 1.10 × 10^−6^ Hz, which was similar to the observed period of about 9–14 days. This indicated that the TRWs might have been trapped by the topography of I1 and I2, as illustrated by the dashed grey curve in Fig. 1b.

As noted by Hamilton^9^, the TRW wavelength is given by


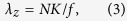


where the trapping depth 1/*λ*_*z*_ can be estimated as the e-folding scale of the TRW amplitude decreasing from the bottom. In this study, we derived it from the first EOF mode of the TRW velocity profiles from August 27, 2012 to August 23, 2013. The results showed that the trapping depth was about 325 m. At the mooring site, *f* was 2.5 × 10^−5^ s^−1^, and *N* was about 10^−3 ^s^−1^. Based on equation (3), the corresponding wavelength was ~82 km. From the inferred TRW characteristics, 
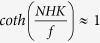
, which agreed with the previous assumption.

### Generation of TRWs

For TRWs having a short period of 9–14 days, their generation must be local because of the restrictive topographic slopes, and they must be trapped near the escarpment^6^,^12^. The power spectra of the deep current at M2 suggested that there was no signal of TRWs even though M2 was separated from M1 by only 40 km (Fig. 5a). This is not surprising because the TRW energy could be inhomogeneous^27^. On the other hand, the exact translation paths of the TRWs cannot be determined using only one mooring. If M1 were downstream of M2, this would suggest that the TRWs observed at M1 were generated locally. Moreover, this could also indicate that the site of M2 was outside the influence of TRWs generated at M1. Because the inferred wavelength of TRWs at M1 was about 82 km, which is larger than the scale of the Rongshu Reef (I1) of ~50 km, the topography associated with the TRWs may be the larger scale feature encompassing both I1 and I2 (Fig. 1b). In other words, the TRWs likely propagated around the topography of I1 and I2, as shown by the dashed grey curve in Fig. 1b.

**Figure 4:**
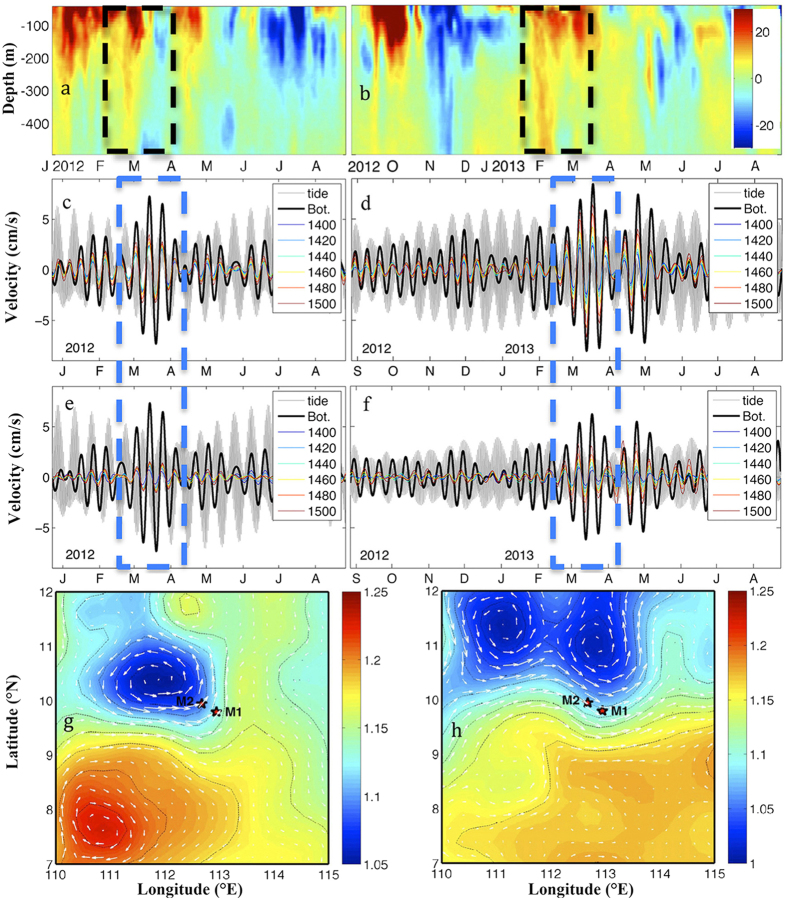
Currents in the upper layers, TRWs, and surface satellite observations. (**a**,**b**) Upper layer zonal velocity profiles at M1 (unit: cm/s); 9–14 day band-pass filtered deep layer (**c**,**d**) zonal and (**e**,**f**) meridional velocities (unit: cm/s); the monthly absolute dynamic topography and surface geophysical current in February (**g**) 2012 and (**h**) 2013. The grey lines are the zonal (**c**,**e**) and meridional (**d**,**f**) tidal currents at 1730 m. Maps were generated by using MATLAB.

Locally TRWs could be generated through the energy transfer from upper-layer eddies to the lower layer by potential vorticity adjustment to the changing depth of the bottom or interface between layers^9^,^28^. There were two large-amplitude TRWs observed in March 2012 and 2013 (Fig. 4c–f). Both TRW events started from February, when the currents in the upper layers obviously strengthened, and the two strong upper layer current events in February 2012 and 2013 reached more than 400 m in depth (Fig. 4a,b). Further analysis of the time-averaged absolutely dynamic topography (similar to the sea level but with respect to the geoid) from Archiving, Validation and Interpretation of Satellite Oceanographic (AVISO) data and the corresponding geostrophic currents at the surface during February 2012 and 2013 indicated that the enhanced upper layer currents were likely associated with cyclonic eddies (Fig. 4g,h). This is consistent with the findings in the Gulf of Mexico that perturbations from eddies in the upper layer, loop current, and meanders are the energy source generating TRWs^3^,^5^,^8^,^9^.

## Discussion

**Figure 5:**
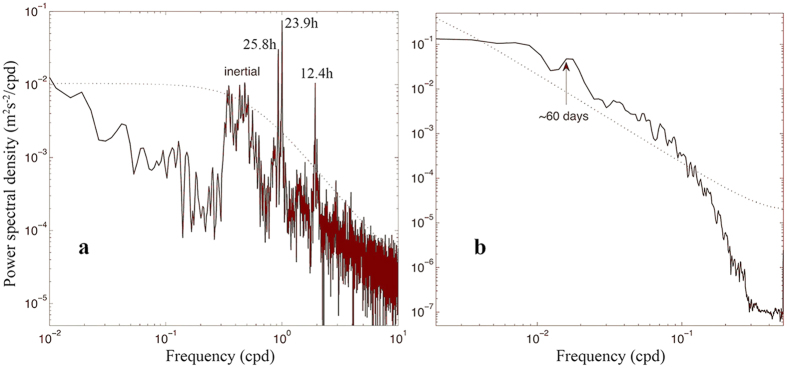
Power spectra of the upper layer velocity at M1 and bottom velocity at M2. (**a**) Power spectrum derived from the near-bottom zonal velocity at M2 observed between August 25, 2013 and September 22, 2014. (**b**) Power spectrum of the vertically averaged zonal velocity between 40 and 450 m at M1 from May 24, 2009 to August 23, 2013, for which a 3-day low-pass filter was used at first. Dotted lines represent a significance level of 95%. Figures were plotted using MATLAB.

As described above, the TRWs were always present in the southern SCS throughout the 5-year observation period, and their amplitudes were comparable to that of tides and much larger than the long-term averaged currents near the bottom, albeit their intensity varied with time. The abrupt intensification of the TRW amplitudes appeared to be due to eddies in the upper layer. In order to answer why these TRWs were able to persist throughout the observation interval, the power spectrum of the depth-averaged, 3-day low-passed, zonal velocity time series above the 450 m depth for all 5years is shown in Fig. 5b. There is an obvious frequency peak with a period of about 2 months. In other words, the upper-layer currents had a strong 2-month oscillation, which could offer an energy source for the TRWs. Moreover, Fig. 4 shows that the TRWs triggered by the upper layer perturbations remained for about 1–2 months in deep layers. Therefore, the TRWs persisted through time.

The SCS topography is very complex and includes four groups of islands: Dongsha, Xisha, Zhongsha and Nansha. The SCS also has a high occurrence of eddies^29^,^30^,^31^,^32^,^33^. Similar to eddies shed from the loop current in the Gulf of Mexico, many eddies originate from the Kuroshio and propagate westwards into the SCS^34^,^35^,^36^,^37^. Moreover, many eddies are generated in the interior SCS^29^,^31^. Together with the complex topography, upper-layer perturbations due to eddies can trigger TRWs with different frequencies in the SCS. We analysed the long-term measurements of a moored Aanderaa current meter (Supplementary Data 1) in the Xisha Islands area and again found fluctuations in the 10–14 day frequency band, which can be attributed to TRWs (Supplementary Figs. 2 and 3).

Hamilton^5^ suggested that 80–90% of the low-frequency velocity variance at depths of greater than 1000 m in the Gulf of Mexico can be explained by TRWs. Similarly, the observed TRWs in the southern SCS indicated that the amplitudes of the TRWs were about one order of magnitude larger than the time-averaged velocity near the bottom at M1. Previous studies speculated that the quick replenishment of the deep SCS water is the result of strong diapycnal mixing associated with the breaking internal tides as they encounter the complex bottom topography^19^,^20^,^21^,^22^. Our results implied that TRWs can have a role comparable to tides in the complex topography area. Of course, we mainly focused on a single mooring, but the observed period was exceptionally long for showing the persistency of the signals. More observations are needed to further quantify the spatial characteristics of TRWs and their contributions to the deep horizontal circulation and overturning circulation.

## Methods

### Measurements from the mooring system

Two moorings (M1 and M2) were deployed in the Nansha Islands region of the southern SCS (red stars in Fig. 1b). M1 was located at 112.96°E 9.79°N, north of the Yongshu Reef, where the water depth is ~1750 m. There were upward- and downward-looking ADCPs at M1 from May 24, 2009 to August 23, 2013, which were redeployed each year. The upward-looking ADCP was generally deployed at ~450 m with a frequency of 75 kHz, and the downward-looking ADCP was at ~1400 m with a frequency of 150 kHz. The upward- and downward-looking ADCPs had sampling time intervals of 1 h and 30 minutes, respectively, and vertical resolutions of 8 and 4 m, respectively. An Aanderaa current-meter was positioned 20 m above the bottom, and it collected valid data from December 23, 2011 to August 26, 2012 and from August 27, 2012 to August 23, 2013. M2 was deployed at 112.66°E 9.98°N, 40 km northwest of M1, from August 25, 2013 to September 22, 2014, where the water depth is about 1800 m. M2 was instrumented with an Aanderaa current-meter 280 m above the seafloor, which had a sampling interval of 1 h. Table 1 presents more details of the moorings.

All data underwent basic quality assurance, during which suspect values were flagged. The valid ADCP data were from a depth of 40 m to the position of the upper ADCP and from the position of the deep ADCP to 120 m below. Short gaps caused by flagged data or redeployment of the mooring were subsequently filled by linear interpolation.

### WOD CTD profiles

Three CTD profiles ~100 km north of M1 and M2 (S1–S3 in Fig. 1b) were obtained from WOD09 to estimate the deep layers’ stratification.

### Sea surface data from satellites

Daily absolutely dynamic topography and surface geostrophic velocities in February 2012 and February 2013 were obtained from AVISO gridded dataset to illustrate the dynamical background at the surface.

## Additional Information

**How to cite this article**: Shu, Y. *et al.* Persistent and energetic bottom-trapped topographic Rossby waves observed in the southern South China Sea. *Sci. Rep.* **6**, 24338; doi: 10.1038/srep24338 (2016).

## Supplementary Material

Supplementary Information

## Figures and Tables

**Table 1:** List of moorings and instruments and their deployment periods.

	Measurement Period	Instrument Type	Measurement Range (m)	Measurement Type	Samplingfrequency
M1 1750 m	20090524–20101109	Upward 75 K ADCP	20–400	Velocity profile	1 hour
		Downward 150 K ADCP	1380–1500	Velocity profile	0.5 hour
	20101110–20111207	Upward 75 K ADCP	20–415	Velocity profile	1 hour
		Downward 150 K ADCP	1400–1480	Velocity profile	0.5 hour
	20111223–20120826	Upward 75 K ADCP	20–500	Velocity profile	1 hour
		Downward 150 K ADCP	1380–1520	Velocity profile	0.5 hour
		Aanderaa RCM11	20 m above the bottom	Velocity, Temperature	0.5 hour
	20120827–20130823	Upward 75 K ADCP	20–500	Velocity profile	1 hour
		Downward 150 K ADCP	1380–1508	Velocity profile	0.5 hour
		Aanderaa RCM11	20 m above the bottom	Velocity, Temperature	1 hour
M2 1800 m	20130825–20140922	Aanderaa RCM11	1520	Velocity, Temperature	1 hour
