# Supplementary material for: Persistent and energetic bottom-trapped topographic Rossby waves observed in the southern South China Sea
Source: Sci Rep. 2016 Apr 14;6:24338. doi: 10.1038/srep24338 (PMC4830981; doi:10.1038/srep24338)
Supplement: Supplementary Information [file srep24338-s1.pdf]

Persistent and energetic bottom-trapped topographic Rossby waves observed in the southern South China Sea

Yeqiang Shu[1,2], Huijie Xue[1,2], Dongxiao Wang[1], Fei Chai[2], Qiang Xie[3], Shuqun Cai[1], Rongyu Chen[1], Ju Chen[1], Jian Li[1], Yunkai He[1]

1, State Key Laboratory of Tropical Oceanography, South China Sea Institute of Oceanology, Chinese Academy of Sciences, Guangzhou, People's Republic of China;

2, School of Marine Sciences, University of Maine, Orono, Maine, USA,

3, Sanya Institute of Deep-Sea Science and Engineering, Chinese Academy of Sciences, Sanya, People's Republic of China.

Corresponding author: Huijie Xue

Address: State Key Laboratory of Tropical Oceanography, Chinese Academy of Sciences, 164 West Xingan Road, Guangzhou 510301, China

Phone: +86 20 89023486

Email: huijiexue@scsio.ac.cn

Supplementary Information

Supplementary Data 1 **|** An Aanderaa current-meter was positioned at 30 m above the bottom at M3 (17.17ºN and 110.43ºE, the Triangle in Fig. 1) in Xisha Islands area where water depth is ~1450 m, and the valid current-meter data were collected from October 2010 to March 2011.

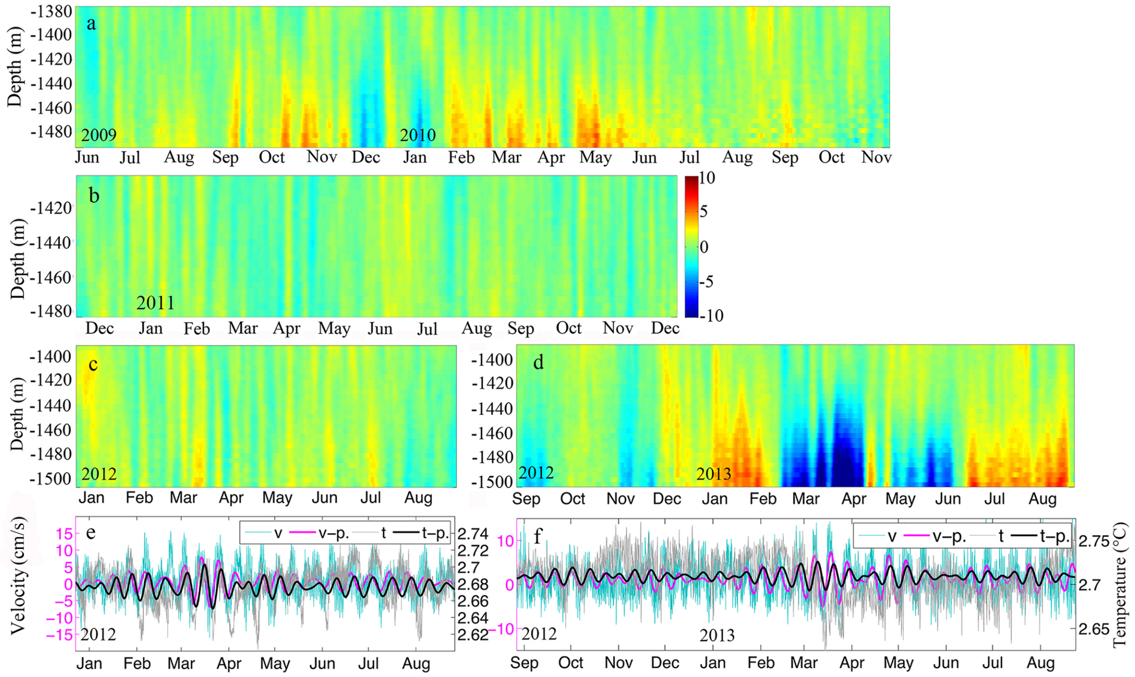

Supplementary Figure 1 **|** Observed near-bottom meridional velocity at M1 (unit: cm/s). (a–d) Colour map representing the four segments of the velocity profiles observed by the downward-looking ADCP. Time series of the meridional velocity (cyan line) and temperature (grey line) obtained by the Aanderaa current meter at 1730 m (20 m above the bottom) (e) from December 23, 2011 to August 26, 2012 and (f) from August 27, 2012 to August 23, 2013. The solid heavy black and magenta lines represent the 9–14 day band-pass filtered meridional velocity and temperature, respectively. Figures were plotted using MATLAB.

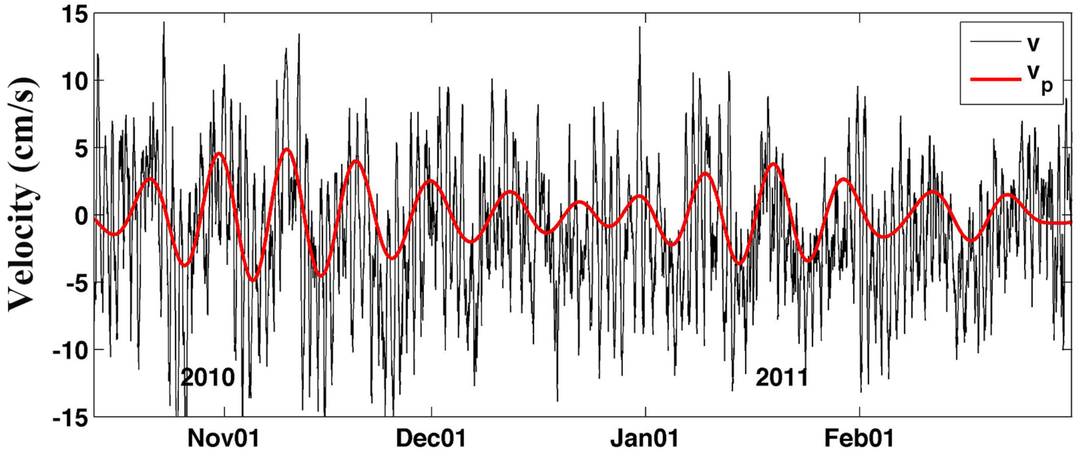

**Supplementary Figure 2 | ** Time series of the meridional velocity (black line) at M3 obtained using Aanderaa current meters at 1420 m (30 m above the bottom) from October 2010 to March 2011, in which the solid heavy red line represents the 9-14 day band-pass filtered meiridioanl velocity. Figure was plotted using MATLAB.

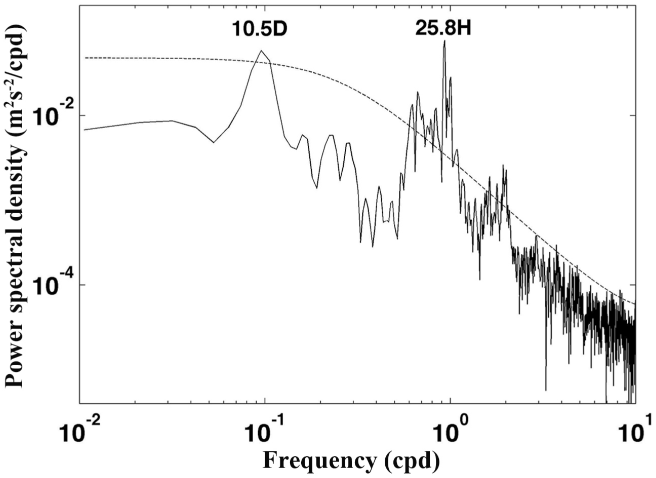

**Supplementary Figure 3 | ** Power spectrum derived from the near bottom meridional velocity at M3 in Xisha Islands area observed between October 2010 and March 2011. Dashed line represents the 95% significance. Figure was plotted using MATLAB.
